# Energy drinks consumption and association factors among medical science students in Syria: A cross-sectional study

**DOI:** 10.1097/MD.0000000000045245

**Published:** 2025-10-17

**Authors:** Moath Salem, Areej Kahwaji, Lugien Al Asadi, Yusra Hourania, Razan Mhawesh, Eiman Nasab, Tamim Alsuliman

**Affiliations:** a Faculty of Medicine, Damascus University, Damascus, Syria; b Faculty of Pharmacy, Damascus University, Damascus, Syria; c National Institute of Health and Medical Research INSERM, UMR 1193, Paris-Saclay University, Paul-Brousse University Hospital, Hepatobiliary Centre, Villejuif, France; d Faculty of medicine, Al Sham Private University, Damascus, Syria; e Service d’Oncologie - Hématologie, Centre Hospitalier de Saint-Quentin, Saint-Quentin, France; f Service d’d’Hématologie Clinique et Thérapie Cellulaire, Hôpital Saint-Antoine, AP-HP, Paris, France.

**Keywords:** energy drinks, medical students, Syrian students

## Abstract

Energy drinks (EDs) are widely marketed as energy boosters, particularly in venues frequented by young people. Despite their association with adverse effects such as elevated blood pressure, increased heart rate, and respiratory issues, EDs remain highly popular, especially among college students seeking to stay awake and focused during exam periods. Limited research has been conducted on ED consumption among Syrian medical students. This study aims to assess the prevalence of ED consumption, knowledge levels, consumption patterns, and associated side effects among medical university students. A cross-sectional study was conducted using a self-administered, structured, and anonymous questionnaire distributed electronically via social media platforms. The survey targeted medical university students, employing a convenience sampling technique to recruit 496 participants. Statistical analysis was performed using R statistical software version 4.3.1. Approximately 30% of participants reported consuming EDs. Of these, 45% consumed EDs daily, while 57% reported consumption 1 to 3 times per week. The main reasons for ED consumption were to gain energy, stay focused, and hydrate when thirsty. Commonly reported adverse effects included insomnia and frequent urination. Females constituted the majority of ED consumers (63%); however, gender was not significantly associated with ED consumption. Bivariate analysis identified attending a private university, living alone, and having a serious health condition as factors associated with ED consumption. Energy drink consumption is prevalent among medical students despite its adverse effects. Educational campaigns promoting healthy dietary habits, addressing misconceptions about EDs, and highlighting their side effects are urgently needed, particularly in medical colleges. Enforcing stricter regulations on the sale of EDs, especially to young individuals, could help regulate consumption, particularly in Syria, where many of these products enter the market illegally due to sanctions.

## 1. Introduction

Energy drink (ED) manufacturers often market their products as “energy boosters” due to the high concentration of legal stimulants they contain. EDs are beverages commonly consumed for their perceived ability to provide a rapid boost in energy, particularly among individuals experiencing fatigue or seeking enhanced performance during physical exercise or competitive sports.^[1]^ The primary ingredient in most of ED is caffeine, ranging from 50 to 180 mg per 8-ounce serving, which is roughly equivalent to 5 ounces of espresso.^[2]^ Additionally, these drinks include sugar and other additives like guarana, taurine, and L-carnitine, which are known to enhance alertness, increase attention and energy, and elevate blood pressure, heart rate, and breathing. Students frequently turn to these beverages for an extra burst of energy.^[3]^ In recent years, manufacturers have shifted their target audience from athletes to younger demographics, particularly teens and young adults. EDs are heavily marketed in venues frequented by these groups. Data show that men aged 18 to 34 years are the highest consumers of EDs, with nearly one-third of teenagers aged 12 to 17 consuming them regularly.^[4]^ Previous reviews have highlighted that boys are more likely to consume energy drinks than girls, with branding and marketing identified as significant influencing factors.^[5]^

A systematic review revealed that tachycardia (elevated heart rate) was the most commonly reported cardiorespiratory event, followed by heart palpitations. Gastrointestinal issues, such as abdominal pain or stomachache, were also frequently observed.^[6]^

Despite the health concerns correlated with the excessive consumption of EDs, it is still on the rise globally, with the United States leading in per capita consumption at 29.69 liters, followed by the United Kingdom at 14.13 liters per capita.^[7]^ Furthermore, a strong positive correlation was identified between ED consumption and behaviors such as smoking, alcohol use, binge drinking, and substance abuse.^[5]^

Medical students, in particular, are subjected to intense academic demands throughout their studies, striving for high grades to secure admission to higher education.^[8]^ The primary reasons for using EDs among this group were staying awake and increasing alertness while studying, along with habitual consumption.^[9,10]^ The growing trend of mixing EDs with alcohol presents a new concern, with evidence indicating that college students who use alcohol mixed energy drinks (AmED) tend to consume alcohol more often than those who do not combine alcohol with EDs,^[11]^ increasing the risk of severe dehydration and alcohol poisoning.^[12]^

The policies for addressing this issue can range from individual school handbook regulations to district-wide bans and even national athletic association guidelines. However, restricting the use of these beverages among university students is challenging, highlighting the need for self-awareness regarding personal consumption.^[13]^ A study by Mohammed et al found that while participants were aware that EDs contain caffeine, they were unaware of other stimulants in the ingredients, and nearly half did not realize that overconsumption could lead to death.^[14]^ Similarly, only half of Saudi adolescents knew that EDs contained caffeine, with most perceiving them as soft drinks.^[15]^ To our knowledge, no prior studies have been conducted on energy drink consumption patterns among Syrians. Therefore, the current study aims to assess the prevalence of ED consumption and the level of knowledge among medical university students, as well as to determine their consumption patterns and the side effects experienced. Additionally, this study investigates the causal relationships between sociodemographic factors, experienced adverse events, and consumption patterns among ED consumers.

## 2. Methods

### 2.1. Study design and data collection

A cross-sectional study was conducted using a self-administered, structured, and anonymous questionnaire to gather data. The questionnaire was distributed electronically via social media platforms, such as Facebook and WhatsApp groups, between October 19, 2022, and October 28, 2022. The only inclusion criterion was that participants be medical students enrolled in any academic year at a Syrian university. A total of 496 students met this criterion, fully completed the questionnaire, and were actively included in the study. The sample size was calculated based on the accessible population, represented by targeted online social groups comprising medical students. A proportion of 15% of the total group membership was utilized, as this percentage represents a scientifically reasonable estimation given the substantial size of the overall group population.^[16]^ A convenience sampling technique was employed, allowing the survey link to be shared among the targeted population and ensuring that all available subjects could be selected, thus improving the representation of the entire population. All collected data were treated with confidentiality and used solely for academic purposes.

### 2.2. Study tool

A validated and reliable questionnaire, developed using Google Forms and adapted from previous literature,^[17–19]^ was employed to meet the study objectives, and tailored to suit the Syrian context. To avoid misunderstandings due to language barriers, the questionnaire was developed and distributed in Arabic. It was initially tested on a small pilot sample to evaluate its accessibility, accuracy, and validity within the target population. The feedback received from this pilot phase was used to further refine the questionnaire. The groups where the questionnaire was posted were educational, and semiofficial online groups. Prior to distribution, the questionnaire underwent a content and relevance validation by university professors to ensure its applicability to the study objectives. Also, the study design was approved by a university committee during their formal evaluation process. The online survey included mandatory response fields to minimize missing data and ensure high response rate. The final questionnaire consisted of 30 questions divided into 3 sections: the first focused on sociodemographic information, the second on ED consumption and related symptoms, and the third on dietary habits and knowledge of certain health recommendations. The estimated completion time for the questionnaire was 5 to 7 minutes. See Questionnaire (Supplemental Digital Content, https://links.lww.com/MD/Q350), which contains all the questions presented to the participants.

### 2.3. Data analysis

Responses were encoded and analyzed using R (R statistical software version 4.3.1, R Foundation). To minimize potential bias, the data analyst blinding method was applied, preventing unconscious manipulation of statistical methods or selective result reporting. Both descriptive and inferential statistics were performed. Descriptive statistics were used to characterize the participants, including frequency, mean, and standard deviation. Missing data was omitted and the remaining data was analyzed. A Chi-square test was conducted to examine the association between ED consumption frequency and sociodemographic factors, drinking habits, and associated effects of EDs. Linear and logistic regression analyses were also performed to evaluate the relationships between variables. A *P*-value of <.05 was considered statistically significant.

## 3. Ethical considerations

All procedures followed the ethical standards of the institutional research committee and the 1964 Helsinki Declaration. Ethical approval was obtained from the Faculty of Medicine, Damascus University, on October 18, 2022. Informed consent was obtained through the first question of the online questionnaire. Participants were required to explicitly agree to participate before being allowed to proceed to the survey questions. Consent was a mandatory condition for inclusion in the study. Additionally, if any participant decided to withdraw at any point during the survey process their data were not included in the final statistical analysis.

## 4. Results

### 4.1. Participants’ characteristics

A total of 496 participants completed the questionnaire. Table 1 provides an overview of the participants’ demographic characteristics and health status. The majority of respondents were female, comprising 318 (64.1%) of the sample, while 178 (35.9%) were male. Most participants were in their fourth academic year (28.6%), followed by those in their second (22.4%) and fifth years (20.6%). A significant portion of the participants resided in Damascus (64.3%), followed by those living in Rif Dimashq (20.2%) and Aleppo (2.8%). Most respondents (74.2%) described their health as good, with 81.0% reporting no underlying diseases and maintaining a healthy weight (mean BMI = 23.1). Only 7.3% of participants lived alone, while the vast majority (82.1%) lived with their families.

**Table 1:** Sociodemographic characteristics of participants (N = 496).

Characteristics	Number of subjects (%)
Gender Male Female	178 (35.9) 318 (64.1)
University Al Baath Aleppo Damascus Hama Tartus Tishreen Other	11 (2.2) 18 (3.6) 217 (43.8) 4 (0.8) 4 (0.8) 9 (1.8) 229 (47.0)
Marital status Single Married Other (engaged, widowed, divorced, etc)	475 (95.8) 7 (1.4) 14 (2.8)
BMI	Mean (SD) 23.1 (6.5)
Academic year 1st 2nd 3rd 4th 5th 6th	23 (4.6) 111 (22.4) 78 (15.7) 142 (28.6) 102 (20.6) 40 (8.1)
College Medicine Dentistry Pharmacy Nursing Health sciences	319 (64.3) 77 (15.5) 71 (14.3) 4 (0.8) 20 (4)
University grade average Fair Good Very good Excellent	29 (5.8) 194 (39.1) 216 (43.5) 57 (11.5)
Country/city of residence Aleppo Damascus Daraa Egypt Hama Homs Iraq Kuwait Lattakia Qamishly Rif Dimashq As Suwayda Tartus	14 (2.8) 319 (64.3) 7 (1.4) 1 (0.2) 4 (0.8) 6 (1.2) 1 (0.2) 1 (0.2) 7 (1.4) 1 (0.2) 100 (20.2) 5 (1.0) 10 (2.0)
With whom do you live Alone Family Friends	36 (7.3) 407 (82.1) 50 (10.1)
Health condition Serious illness Stable Good	12 (2.4) 116 (23.4) 368 (74.2)
Do you suffer from any chronic illness mentioned below Asthma Diabetes Hypertension I don’t Other	30 (6.0) 4 (0.8) 6 (1.2) 402 (81.0) 54 (10.9)

### 4.2. ED consumption and patterns

Table 2 summarizes the ED consumption patterns among the participants. Of the total sample, 149 (30.0%) reported consuming EDs. Nearly half of these consumers (45.0%) drank EDs daily, and approximately 57.0% consumed EDs 1 to 3 times per week. The primary reasons for consumption were to gain energy (30.9%), to stay focused (24.8%), and to hydrate when thirsty (22.8%). A total of 132 participants reported drinking EDs at home, while 79 consumed them at a restaurant or cafe.

**Table 2:** Patterns of ED consumption among medical students.

Characteristics	Number of subjects (%)
Do you consume energy drinks? Yes No	149 (30.0) 347 (70.0)
Age when first started consuming energy drinks	Mean (SD) 15.3 (3.5)
Do you still consume energy drinks till now? Yes No	129 (86.6) 20 (13.4)
What is your drinking frequency? Daily Weekly On occasions only	67 (45.0) 34 (22.8) 48 (32.2)
How many times do you drink per week? 1 to 3 times 4 to 7 times More than 8 times	85 (57.0) 37 (24.8) 27 (18.1)
Reason for drinking energy drinks Alcohol party To stay focused To get high To stay awake To gain more energy To get hydrated when feeling thirsty	12 (8.1) 37 (24.8) 8 (5.4) 12 (8.1) 46 (30.9) 34 (22.8)
Places you consume energy drinks at Home cafe/restaurant/nightclub University Friends’ place School Public places (e.g. garden, beach, etc) Other	132 (88.6) 79 (53.0) 78 (52.3) 88 (59.1) 13 (8.7) 52 (16.1) 24 (16.1)
Have you ever been told to stop drinking? Family Friends Other No	44 (29.5) 6 (4.0) 4 (2.7) 95 (63.8)

ED = energy drink.

### 4.3. Adverse effects, dietary habits, and knowledge of EDs

Among ED consumers, 41.6% reported experiencing adverse effects. As shown in Table 3, the most frequently reported symptoms were insomnia (45%) and frequent urination (41.6%), followed by headaches (39.6%), increased heart rate (38.3%), and tremors (29.5%). Table 4 summarizes participants’ dietary habits, 59.1% of participants consume less than 3 meals per day, while 29.5% eat 3 meals daily. Notably, 44.3% of students eat breakfast most days, 60.4% have 1 to 2 snacks per day, and 62.4% eat 1 to 2 servings of fruits or vegetables daily. The participants’ knowledge regarding EDs was generally poor, with more than 2-thirds being unaware of whether EDs are safe to consume during dehydration or while taking other medications.

**Table 3:** Undesirable effects experienced from ED consumption among participants.

Side effects experienced	Number of subjects (%)
Increased heart rate	57 (38.3)
Headaches	59 (39.6)
Upset stomach	32 (21.5)
Vomiting	8 (5.4)
Tremors	44 (29.5)
Discomfort	28 (18.8)
Erythema	14 (9.4)
Itching	7 (4.7)
Feeling hot	20 (13.4)
Dehydration	26 (17.4)
Insomnia	67 (45.0)
Frequent urination	62 (41.6)
Other	17 (11.4)

ED = energy drink.

**Table 4:** Participants’ dietary habits.

Dietary pattern	Number of subjects (%)
Number of meals consumed daily 3 meals More than 3 <3	44 (29.5) 17 (11.4) 88 (59.1)
Eating breakfast daily Always Mostly Rarely Never	30 (20.1) 66 (44.3) 43 (28.9) 10 (6.7)
Number of snacks consumed daily 1–2 3 or more Rarely	90 (60.4) 16 (10.7) 43 (28.9)
Number of veggie fruit snacks consume daily 1–2 3 or more Rarely	93 (62.4) 12 (8.1) 44 (29.5)
Number of veggie fruit snacks consume weekly 1–2 3 or more Rarely	39 (26.2) 92 (61.7) 18 (12.1)

### 4.4. Sociodemographic factors and consumption patterns

**Figure 1.**
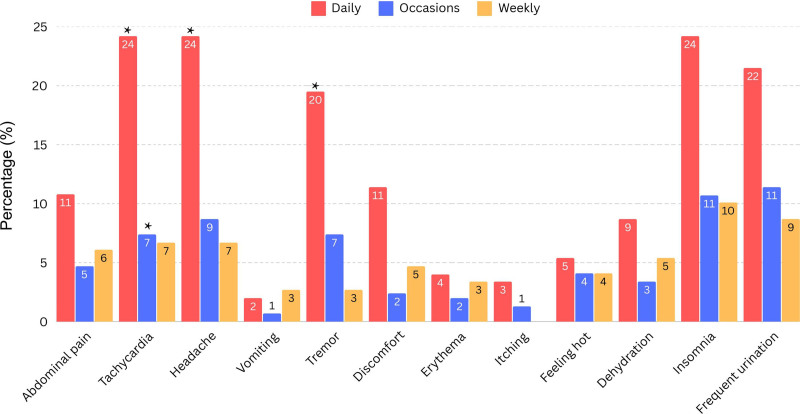
Drinking frequency and associated adverse events. *Significant *P*-value below .05.

Significant associations were identified between ED consumption and certain sociodemographic factors, including attending a private university (*P* < .001), living alone (*P* < .001), and having serious illness (*P* = .01). However, caution is advised when interpreting the latter 2 factors due to the substantial difference in observations. Detailed information is summarized in Table 5. Additionally, a strong correlation was observed between daily ED consumption and 3 specific adverse effects: increased heart rate, headaches, and tremors (Fig. 1). In contrast, other adverse effects reported by participants did not demonstrate a statistically significant association with ED consumption.

**Table 5:** Sociodemographic factors and their association with ED consumption among students who consume ED.

	Do you consume energy drinks?		*P*-value
	No n (%)	Yes n (%)	
What is your university? Public institute Private institute	179 (40.1) 126 (28.3)	38 (8.5) 103 (23.1)	.00
Sex Male Female	123 (24.8) 224 (45.2)	55 (11.1) 94 (19)	.83
With whom do you live? Alone Family Friends	18 (3.7) 287 (58.2) 40 (8.1)	18 (3.7) 120 (24.3) 10 (2.0)	.01
How is your health condition? Serious Stable Good	7 (1.4) 69 (13.9) 271 (54.6)	5 (1.0) 47 (9.5) 97 (19.6)	.01
What is your marital status? Single Married Other	334 (67.3) 5 (1) 8 (1.6)	141 (28.4) 2 (0.4) 6 (1.2)	.56
What is your college? Dentistry Health Sciences Medicine Nursing Pharmacy	56 (11.3) 18 (3.6) 219 (44.2) 2 (0.4) 50 (10.1)	21 (4.2) 2 (0.4) 100 (20.2) 2 (0.4) 21 (4.2)	.2
What is your college degree level? Fair Good Very good Excellent	15 (3) 136 (27.4) 157 (31.7) 39 (7.9)	14 (2.8) 58 (11.7) 59 (11.9) 18 (3.6)	.14

ED = energy drink.

## 5. Discussion

EDs are globally marketed as energy boosters, appealing to individuals who exercise, feel fatigued, or believe caffeine enhances performance in activities such as competitive sports.^[1]^ In our study, approximately 30% of participants reported consuming EDs, a lower proportion compared to 50% among medical students at a Saudi university^[20]^ and 45% among university students in Beirut.^[21]^ This disparity could be attributed to factors such as the relatively high cost of ED products, which many medical students may find unaffordable, and the limited availability of certain imported brands due to international sanctions restricting legitimate importation.

The age group most commonly starting ED consumption in our sample was 12 to 19 years, aligning with global data showing that the 13 to 16 age range is the most prevalent for initiating ED use. This age bracket is particularly critical, as individuals who begin consuming EDs during this period are 4.88 times more likely to consume large quantities compared to those who start between the ages of 20 to 23.^[22]^ However, it is important to note that the American Academy Of Pediatrics strongly advises against ED consumption for children aged 12 to 18, emphasizing the potential health risks associated with these beverages.^[23]^

Interestingly, our findings revealed that females constituted the majority of ED consumers, accounting for 63% compared to 37% for males. This finding challenges the global trend, where males are typically reported as the dominant consumers of EDs. A systematic review highlighted that ED consumption is significantly higher among adolescents and young adults, with boys outpacing girls.^[24]^ Similarly, a 3-year study conducted in Norway reported an increase in ED consumption, rising from 3.3% to 4.9% among females and from 9.8% to 11.5% among males.^[25]^

A notable 45% of participants reported consuming EDs daily, with 57% consuming them 1 to 3 times daily. These figures are relatively high compared to other countries: for instance, daily consumption rates were reported at 4.7% among men and 3.3% among women in Norway,^[26]^ 1.1% among 23,610 students in Canada,^[27]^ and 11.4% in a smaller study of 919 participants in the United Arab Emirates.^[28]^ However, a meta-analysis by Aonso-Diego et al estimated that global daily ED usage could reach as high as 44.8%,^[24]^ aligning more closely with our findings.

Daily consumption of EDs is linked to a variety of adverse effects. For example, insomnia was reported in 51% of women who consumed EDs daily, compared to 33% of women who rarely or never consumed them.^[26]^ A study conducted at the Copperbelt University School of Medicine in Zambia found a statistically significant association between ED consumption and poor sleep quality (*P* < .01), with most students consuming these beverages during exam preparation periods.^[29]^ Likewise, A study involving adolescents and young athletes in Cyprus reported that the most commonly observed adverse effects included insomnia, palpitations, diuresis, headaches, and elevated stress levels.^[30]^ In our sample, insomnia and frequent urination were the most prevalent symptoms, while headaches (39.6%) and increased heart rate (38.3%) were also significant. Symptoms of caffeine intoxication typically occur at doses of 200 mg or higher,^[2]^ and most EDs contain 100 to 300 mg of caffeine per serving.^[31]^ A large national survey of college and university students in Norway revealed that daily ED consumers slept approximately 30 minutes less per night than occasional or non-consumers, with increased nocturnal wakefulness and poorer sleep efficiency.^[26]^

It is well-documented that students, particularly those in medical fields, consume EDs to stay awake and enhance focus during intensive study periods. Our findings align with this, as 30.9% of participants consumed EDs to gain energy and 24.8% to maintain focus while studying. Similarly, a study at the Medical University of Lublin in Poland reported that 75% of medical students consumed EDs to combat sleepiness, with additional reasons including improving mental agility and concentration.^[32]^

Although alternatives like green tea, yerba mate, coffee, and matcha tea have been suggested, these options often lack the flavor appeal and high caffeine content that attracts ED consumers.^[33]^ To address the health risks associated with ED consumption, particularly among vulnerable populations, a U.S. FDA study proposed increased regulatory scrutiny and improved labeling as cost-effective mitigation strategies.^[34]^

Females were found to consume more ED than males in our study; however, no significant correlation was observed between gender and ED consumption when applying the chi-square test (*P* = .83). This finding contrasts with prevailing research indicating that males are significantly more likely to consume EDs compared to females.^[21,35,36]^ After controlling for confounding factors, our results revealed significant correlations between ED consumption and living alone, as well as having a compromised body status.

The annual fees for private universities are substantial compared to the average income in Syria. This being said, affiliation with private universities emerged as a notable variable in our sample. This may be attributed to factors such as the higher cost of EDs, which public university students may find less affordable, and the tendency of private university students to emulate their peers, a behavior that may be more pronounced in significantly costly settings. Additionally, physical activity plays a role in ED consumption. Previous studies have shown that physically active individuals are 1.89 times more likely to consume EDs compared to their inactive counterparts.^[21]^ While the precise biological mechanisms of caffeine remain unclear, it is known to enhance performance by reducing feelings of exhaustion, improving physical endurance, and increasing central drive.^[37]^ Interestingly, research suggests that the odds of ED consumption increase by 31% for every additional instance of viewing extreme sports per week.^[38]^

In light of these findings, it is essential to raise awareness among university students, adolescents, and young adults about the health risks associated with ED consumption. Educational efforts should focus on promoting accurate knowledge of caffeine content, recognizing early signs of overconsumption, and understanding the potential short- and long-term consequences. Students should be encouraged to seek healthier alternatives for boosting energy and concentration, such as proper sleep hygiene, balanced nutrition, and physical activity. Additionally, it is important for this age group to be cautious of excessive reliance on EDs during academic stress or exam periods and to avoid mixing them with other stimulants or alcohol.

## 6. Study limitations

Several limitations of this study warrant discussion. Its cross-sectional design restricts the ability to infer causal relationships between ED consumption and the reported adverse effects. Moreover, important variables such as physical activity, affordability, and detailed attitudes toward EDs were not explored. The reliance on self-reported data introduces the potential for recall bias and social desirability bias, which could affect the accuracy of reported consumption frequencies. Furthermore, the study’s questionnaire was distributed primarily via social platforms including students residing in Damascus, limiting the generalizability of findings to young adults across the country. Due to significant accessibility challenges, particularly in conflict-affected regions, on-site data collection was not feasible during the study period. Political and security restrictions made social media one of the few viable means for reaching a broad segment of Syrian medical students. Additionally, the ongoing instability in the country has led to a lack of publicly accessible datasets from government entities, NGOs, or academic institutions. This absence of national-level data further hindered our ability to obtain a more comprehensive and representative demographic profile of the target population. As a result, future research involving broader geographic inclusion is essential to improve the representativeness and generalizability of the findings. Lastly, conducting additional validation of the questionnaire was not feasible under the given circumstances. Nonetheless, to mitigate this limitation, the Arabic translation of the questionnaire was reviewed by field experts and formally evaluated by the university’s academic committee as part of the approval process.

## 7. Conclusion

ED consumption among medical students remains a significant concern, warranting immediate attention from both educational and public health authorities. There is a clear need for awareness campaigns within medical colleges to promote healthier dietary behaviors, address common misconceptions surrounding EDs, and emphasize their potential health risks. Furthermore, implementing stricter regulations on the marketing and sale of EDs, particularly to young individuals, may help control their widespread availability. This is especially relevant in the Syrian context, where many ED products enter the market through informal channels due to ongoing sanctions and limited regulatory enforcement.

## Author contributions

**Conceptualization:** Moath Salem, Yusra Hourania, Eiman Nasab.

**Data curation:** Moath Salem, Yusra Hourania, Razan Mhawesh, Eiman Nasab.

**Formal analysis:** Lugien Al Asadi.

**Methodology:** Lugien Al Asadi.

**Project administration:** Moath Salem, Areej Kahwaji, Tamim Alsuliman.

**Resources:** Areej Kahwaji, Razan Mhawesh.

**Supervision:** Lugien Al Asadi, Tamim Alsuliman.

**Validation:** Tamim Alsuliman.

**Visualization:** Areej Kahwaji, Tamim Alsuliman.

**Writing – original draft:** Areej Kahwaji.

**Writing – review & editing:** Lugien Al Asadi, Tamim Alsuliman.

## Supplementary Material


