# Supplementary material for: Energy drinks consumption and association factors among medical science students in Syria: A cross-sectional study
Source: Medicine (Baltimore). 2025 Oct 17;104(42):e45245. doi: 10.1097/MD.0000000000045245 (PMC12537174; doi:10.1097/MD.0000000000045245)
Supplement: Supplementary file 1 [file medi-104-e45245-s001.docx]

**The impact of energy drinks on students of medical colleges in Syria: cross-sectional study**

1. Do you agree to participate in the research “*The impact of energy drinks on students of medical colleges in Syria cross-sectional study*”

Yes

No

***Note****: I consent that when filling out this questionnaire, I volunteer to participate in the study, I have no problem using this information, and publishing it for scientific research reasons. I am aware that my personal information will be displayed only with my consent.*

1. University

Damascus University

Aleppo University

Al Baath University

Tishreen University

Tartous University

Hamah University

Other (example: private university)

1. Sex

Male

Female

1. Weight (kg):

______

1. Height (cm):

___

1. Social situation

Single

Married

Other

1. Academic year

1st year

2nd year

3rd year

4rth year

5th year

6th year

1. College

Check all that apply.

Medicine

Dentistry

Pharmacy

Nursing

Health Sciences

1. Age group

18 – 20 years

21 – 22 years

23 – 25 years

26 years or above

1. What is your Academic Grade?

Fair

Good

Very good

Excellent

1. Where do you live currently?

_________

1. With whom do you live currently?

_________

1. How is your health condition?

Good

Stable

Serious illness

1. Do you suffer from one of the following chronic diseases?

Diabetes

High blood pressure

Asthma

I do not suffer from any diseases

Other

1. Do you consume energy drinks?

Yes

No

****If you are a consumer of energy drinks, complete the questionnaire***

16. How old were you when you started consuming energy drinks write down the age.
_________

17. Do you still consume soft drinks till now?

Yes

No

1. How often do you consume energy drinks?

Daily

Weekly

Only on occasions

1. How many times you consume energy drinks per week?

1-3 times

4-7 times

More than 7

- 1. What is your motivation to consume energy drinks?

Lack of sleep

To get hydrated when thirsty

The need for more energy

To help me focus

For more excitement and happiness

When drinking alcohol during parties

1. Where do you consume energy drinks?

At home

At a restaurant, coffee shop or club with friends

In public places: public park, beach, etc

University

At school

Other place

- 1. Has anyone asked you to reduce energy drinks consumption?

Parents

Friends

Someone else

No

- 1. Have you experienced any symptoms as a result of consuming these drinks

Yes

No

- 1. If your answer to the previous question was yes, check the symptom you experience

YES NO

Increased heart rate □ □

Headache □ □

Abdominal pain □ □

Vomiting □ □

Tremors □ □

Discomfort □ □

Erythema □ □

Itching □ □

Feeling hot □ □

Dehydration □ □

Insomnia □ □

Frequent urination □ □

Other symptoms □ □

- 1. How many main meals do you eat per day?

Only 3

Less than 3

More than 3

- 1. Do eat breakfast everyday?

Always

Mostly

Rarely

Never

- 1. How many snacks per day do you eat?

1-2

3 or more

Rarely

- 1. How many veggie fruit snacks do you eat daily?

1-2

3 or more

Rarely

- 1. How many veggie fruit snacks do you eat per week?

1-2

3 or more

Rarely

- 1. What are the recommendations of the specialized health committees regarding the consumption of energy drinks in the following cases: Dehydration after sports and exercises?

Can be consumed

It is forbidden

I don't know

31. Athletes taking other medicines: What are the recommendations of the specialized health committees regarding consuming of energy drinks in this case?

Can be consumed

It is forbidden

I don't know
